# Development of an Intelligent Clinical Decision Support System for Predicting One-Year CPAP Adherence in Patients with Obstructive Sleep Apnea: A Pilot Study

**DOI:** 10.3390/jcm15156073

**Published:** 2026-08-04

**Authors:** Emma López-Prado, Manuel Casal-Guisande, Mar Mosteiro-Añón, Jorge Cerqueiro-Pequeño, Alberto Fernández-Villar, María Torres-Durán

**Affiliations:** 1 School of Industrial Engineering, University of Vigo, 36310 Vigo, Spain; emma.lopez.prado@alumnado.uvigo.gal (E.L.-P.); mar.mosteiro.anon@sergas.es (M.M.-A.); 2 Department of Design in Engineering, University of Vigo, 36208 Vigo, Spain; jcerquei@uvigo.gal; 3 NeumoVigo I+i Research Group, Galicia Sur Health Research Institute (IIS Galicia Sur), SERGAS-UVIGO, 36312 Vigo, Spain; jose.alberto.fernandez.villar@sergas.es (A.F.-V.); maria.luisa.torres.duran@sergas.es (M.T.-D.); 4 Centro de Investigación Biomédica en Red, CIBERES ISCIII, 28029 Madrid, Spain; 5 Pulmonary Department, Hospital Álvaro Cunqueiro, 36312 Vigo, Spain; 6 Department of Functional Biology and Health Sciences, University of Vigo, 36310 Vigo, Spain

**Keywords:** obstructive sleep apnea, CPAP, adherence, design, machine learning, clinical decision support system

## Abstract

**Background/Objectives:** Obstructive sleep apnea (OSA) is a prevalent chronic disorder whose first-line treatment, continuous positive airway pressure (CPAP), is effective only if the patient maintains sufficient adherence. Early predictions of the risk of low adherence would make it possible to personalize follow-up and optimize healthcare resources. The aim of this study was to develop and evaluate a machine-learning-based clinical decision support system to predict CPAP adherence after one year of treatment. **Methods:** A cohort of 200 patients with OSA from the Sleep-Disordered Breathing Unit of Hospital Álvaro Cunqueiro in Vigo was used. The cohort was split into a training set (*n* = 160) and an independent test set (*n* = 40). Two scenarios were defined: Scenario A, with pre-treatment variables, and Scenario B, which also includes early adherence metrics. In each scenario, variables were selected through recursive feature elimination. The selected variables were apnea-hypopnea index (AHI), 3% oxygen desaturation index (ODI3%), chronic obstructive pulmonary disease and neck circumference in Scenario A, and first-month adherence, ODI3% and AHI in Scenario B. Once these subsets were defined, several classifiers were analyzed. **Results:** Random Forest was the model selected in both scenarios. On the test set, Scenario A reached an area under the curve (AUC) of 0.71 (sensitivity 0.83; specificity 0.45) and Scenario B an AUC of 0.91 (sensitivity 0.90; specificity 0.82). **Conclusions:** Early prediction of CPAP adherence using machine learning is feasible; incorporating real first-month use markedly improves discriminative ability. The system was integrated into a web prototype as a proof of concept. Given the modest sample size and the absence of external validation, these results should be interpreted as preliminary, corresponding to an exploratory, feasibility study. For future implementation, an extensive clinical validation process will be required, along with the expansion of the database, which will likely contribute to improving the system’s robustness and generalization capacity.

## 1. Introduction

Obstructive sleep apnea (OSA) is a chronic respiratory disorder characterized by repeated episodes of upper-airway collapse during sleep, which fragment rest and cause repeated oxygen desaturations. Its effects go beyond daytime sleepiness, as it is associated with arterial hypertension and with cardiovascular and cerebrovascular disease, and it multiplies the risk of traffic accidents, making it a public-health problem with an impact on the healthcare system [1]. The accident risk in untreated patients is estimated at 2.4 times that of the general population, reaching 3 to 7 times in severe cases [2,3]. In Spain, OSA is estimated to affect several million people, although the exact burden depends on the diagnostic definition and severity threshold used [4]. Previous Spanish estimates reported that approximately 1.2–2.15 million people may have OSA, with only 5–9% being diagnosed [5]. Underdiagnosis remains particularly relevant, especially among women, with population-based data suggesting that up to 93% of women with moderate-to-severe sleep apnea may remain clinically undiagnosed [6]. This hidden burden is clinically relevant, since OSA is strongly associated with cardiometabolic and neurocognitive comorbidity. Metabolic syndrome is highly prevalent among adults with polysomnography-confirmed OSA, with recent meta-analytic evidence estimating a pooled prevalence of 55.4% [7]. Hypertension is one of the most closely related cardiovascular conditions, affecting approximately 50% of patients with OSA, while OSA may be present in up to 80% of patients with resistant hypertension [8]. Severe untreated OSA has also been associated with increased mortality and cardiovascular risk, including ischemic heart disease, heart failure, arrhythmias and stroke [9,10,11,12,13]. In addition, sleep-disordered breathing has been linked to a 26% higher risk of developing cognitive impairment [14]. Among the main risk factors, obesity plays a central role, together with age and male sex [14].

OSA also entails an economic and healthcare burden. The direct healthcare costs associated with the untreated disease in Spain have been estimated at more than EUR 5 billion per year, and sleep units manage a high and growing volume of patients on CPAP treatment. When there is clinical suspicion, diagnosis requires a sleep study: although polysomnography is the reference test, home respiratory polygraphy is the usual technique in routine care. OSA is confirmed when the apnea-hypopnea index (AHI) is ≥15 events/hour, or ≥5 accompanied by related clinical symptoms [1].

The reference treatment is continuous positive airway pressure (CPAP), which keeps the upper airway open, reduces respiratory events and improves oxygenation and daytime sleepiness [15]. However, its benefit depends on adherence; a patient is considered adherent when they use the device at least 4 h per night on ≥70% of nights [16], a threshold that between 46% and 83% of patients do not reach [17]. The underlying problem is the difficulty of anticipating which patients will maintain adequate adherence, since this is confirmed only after prolonged follow-up and depends on multiple factors—e.g., OSA severity, tolerance, perceived benefit, comorbidities and initial use—that are rarely integrated into an individualized estimate of the risk of treatment discontinuation or low use.

In this context, artificial intelligence (AI) tools, and in particular machine-learning-based approaches, constitute a promising way to assist the professional in risk stratification and the selection of interventions [18,19,20,21,22,23,24,25,26,27,28]. The literature agrees that predicting adherence based only on baseline variables has limited power and that incorporating early markers of real use improves the predictive performance of these systems [29].

On these premises, the main objective of this work is to develop and evaluate an intelligent clinical decision support system capable of predicting one-year CPAP adherence in patients with OSA.

## 2. Materials and Methods

### 2.1. Cohort Description

A retrospective, single-center study was conducted using a clinical database from the Sleep-Disordered Breathing Unit (SDBU) of Hospital Álvaro Cunqueiro in Vigo, which contains information on approximately 10,000 patients referred for suspected OSA. The use of these data for research purposes was approved by the Research Ethics Committee of Galicia (code 2022/256).

After removing duplicate records, only patients with an indication for CPAP treatment during 2024 and with available one-year follow-up adherence data were selected. After applying these inclusion criteria, the final sample comprised 200 patients, for whom multiple clinical, demographic and treatment-related variables were collected. The information available for each patient was grouped into the following blocks:

- Demographic and anthropometric data: Sex, age, body mass index (BMI), neck circumference, work shift and professional-driver status.
- Clinical and pharmacological information: Comorbidities (hypertension, stroke, diabetes, ischemic heart disease, atrial fibrillation, heart failure, chronic obstructive pulmonary disease (COPD), rhinitis, depression) and drugs (antidepressants, antihistamines).
- Symptoms: Daytime tiredness, morning grogginess, awakenings, witnessed apneas, sleepiness, the Epworth scale and nocturia, among others.
- Sleep metrics: Apnea-hypopnea index (AHI), supine and lateral AHI, manual 3% oxygen desaturation index (ODI3%), percentage of time with oxygen saturation below 90% (TC90), and counts of obstructive, central and mixed apneas and hypopneas.
- CPAP settings: Initial pressure (fixed or automatic at the 90th percentile) and humidification.

The cohort has a mean age of 55.97 ± 12.11 years, a predominance of men (75.0%), a BMI of 33.46 ± 6.23 kg/m^2^ and an AHI of 41.72 ± 23.48 events per hour, reflecting a sample with high obesity and severity (Table 1). The target variable is one-year adherence, coded in binary form (1 = adherent; 0 = non-adherent) according to the criterion of use ≥4 h/night on ≥70% of nights [16]: 145 patients were adherent and 55 non-adherent (a rate of 72.5%).

To explore the predictive value of early adherence, two scenarios were defined: Scenario A, with clinical, demographic, anthropometric and symptom variables and diagnostic-test variables collected before treatment; and Scenario B, which adds first-month adherence. First-month adherence was defined using the same composite criterion as the target variable (use ≥4 h/night on ≥70% of nights during the first month) and was coded in binary form (adherent/non-adherent). A total of 20% of the patients were reserved as an independent test set and the remaining 80% for training and tuning, using a partition stratified by the target variable, fixed and identical for both scenarios to ensure comparability (Table 2). The test set was kept isolated throughout the entire development and reserved for a single final evaluation.

### 2.2. Quality Control and Outliers

Quality control was performed during preprocessing. In addition to removing duplicates and applying the clinical and temporal filters, the variants of the tobacco and alcohol consumption variables were homogenized, the work shift was recoded into three categories (“No shift”, “Exempt from work” and “Shifts”), and constant variables were discarded. Missing values were handled with two strategies:

- Record removal in the essential variables (AHI, TC90, Epworth, BMI, sex, age and main symptoms).
- Imputation by clinical reference value in variables with a low rate of missing data: in agreement with the SDBU service, it was considered that, in these variables, the missing data actually corresponded to the “No” category, and were therefore imputed as such. This imputation was restricted to variables with a very low proportion of missing values and was based on a clinical criterion agreed upon with the unit; nonetheless, it is assumed to be a simplification that could introduce some bias, and it is therefore explicitly acknowledged as a limitation of the study.

Outliers were detected in the continuous numeric variables using the interquartile range (IQR), considering as an outlier any value outside the interval (Q1 − 1.5 × IQR, Q3 + 1.5 × IQR). The highest proportions corresponded to the count of respiratory events (mixed apneas 13.0%, central 11.5%, obstructive 6.0%); these values were kept as they correspond to plausible clinical profiles.

### 2.3. Conceptual Design of the Intelligent System

**Figure 1:**
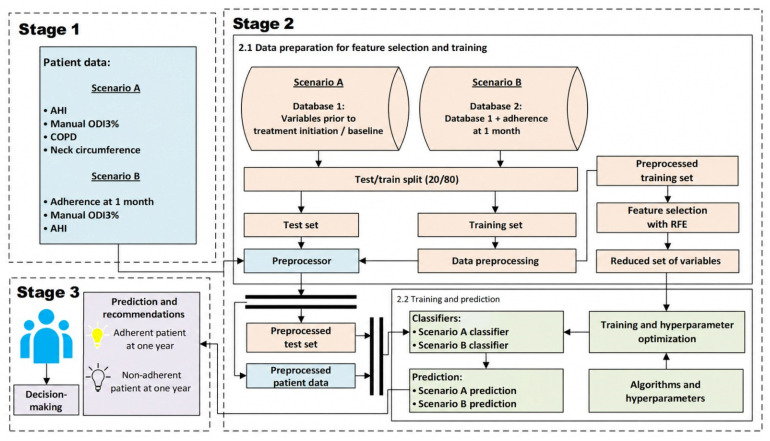
Conceptual design of the intelligent clinical decision support system, organized in three stages: data collection and preprocessing, processing (feature selection, training and prediction), and evaluation, presentation and decision support.

The system is conceived as an intelligent clinical decision support system that, based on a patient’s data, estimates their probability of one-year CPAP adherence and conveys the result to the professional. Its operation is structured in three stages (see Figure 1): (1) collection and preprocessing of the patient’s data; (2) processing by the intelligent system, with a preparation and feature-selection phase and a training and prediction phase; and (3) evaluation, presentation of the result and decision support. In this last stage, a probability estimated by the model and a binary classification derived from the decision threshold are obtained. If the patient is classified as adherent, no alert is triggered and routine follow-up is maintained; if classified as non-adherent, the system triggers an alert and generates indicative recommendations (addressing barriers, reinforcing therapeutic education, nursing intervention or intensified follow-up). In any case, the tool does not replace the assessment of the healthcare professional.

### 2.4. Implementation of the Machine Learning System

All modeling was implemented in MATLAB (version R2025b, Natick, MA, USA). Numeric variables were normalized by standardization (z = (x − μ)/σ), estimating μ and σ on the training set; binary variables were coded as 0/1 and categorical variables with more than two categories using one-hot encoding [30]. Once the models were developed, the application prototype was implemented in Python (version 3.13) using the Streamlit library.

#### 2.4.1. Feature Selection

Among filter, wrapper and embedded methods [31], a wrapper method of recursive feature elimination (RFE) was used with a Random Forest (RF) of 200 trees and out-of-bag (OOB) prediction as the estimator, given its tolerance to collinearity and to the nonlinear relationships that may be present in clinical data [30]. The process, run independently per scenario and on the training set, consisted of:

1. Permutation importance calculation: The values of each predictor are permuted in the OOB observations and the increase in classification error is measured; the greater the increase, the greater the relevance. Starting from the complete set of variables, the variables are then eliminated one at a time in successive iterations.
2. Iterative elimination of the least relevant predictor (step of one), exploring nested subsets from the full set down to a single predictor.
3. Evaluation of each subset by stratified five-fold cross-validation (CV-5), concatenating the out-of-fold (OOF) scores to obtain an AUC per subset and to build the AUC profile against the number of variables.
4. Informed manual selection: a reduced subset of variables was selected for each scenario based on its AUC, with the aim of favoring clinical interpretability.

**Figure 2:**
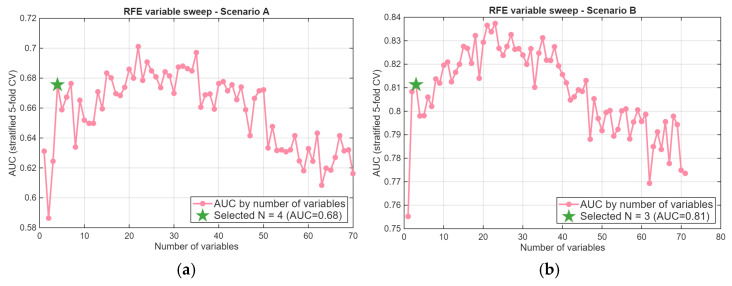
Evolution of the cross-validation AUC according to the number of variables during RFE. (**a**) Scenario A; (**b**) scenario B.

The AUC profiles are shown in Figure 2 and the selected variables in Table 3 for each of the scenarios.

#### 2.4.2. Training, Decision Threshold and Evaluation

Once the subset of predictor variables was obtained, four classifiers were trained and optimized: Random Forest (RF), logistic regression with Lasso penalization (LR), support vector machine with radial kernel (SVM-RBF) and Gradient Boosting (GB). For each one, a set of hyperparameter configurations was explored using stratified CV-5 on the training set, with the mean AUC as the criterion. The hyperparameter spaces were as follows:

- RF: Number of trees ∈ {100, 200, 300}; minimum leaf size ∈ {3, 5, 10, 20}.
- LR (Lasso): λ over 20 logarithmic values between 10^−4^ and 10^1^.
- SVM-RBF: C ∈ {0.01, 0.1, 1, 10, 100} and γ ∈ {0.001, 0.01, 0.1, 1}.
- GB: Number of cycles ∈ {50, 100, 200}; learning rate ∈ {0.05, 0.1, 0.2}; depth ∈ {2, 3, 5}.

The classifier with the highest mean AUC in cross-validation was designated the representative model for each scenario. Model and hyperparameter selection were based on cross-validation on the training set, and the test set did not take part in the modeling. The decision threshold was obtained from the OOF scores by maximizing the Matthews Correlation Coefficient (MCC), chosen because it provides a balanced evaluation in problems with imbalanced classes such as our database [32,33], and was fixed for its application on the test set. Finally, the representative model was applied only once to the test set. For the metrics (AUC, sensitivity and specificity), 95% confidence intervals were computed by percentile bootstrap with 2000 resamples. For the Random Forest, the number of trees and the minimum leaf size were tuned by grid search with cross-validation; the remaining parameters (the number of predictors sampled at each split and the split criterion) were kept at their default values; in addition, the trees were grown without a depth limit, controlled solely by the minimum leaf size. Class imbalance was not addressed through class weighting but through the decision threshold optimized by the MCC.

#### 2.4.3. Model Selection

Table 4 shows the training and selection results in each scenario (mean cross-validation AUC and sensitivity/specificity with the MCC threshold on the OOF scores). In Scenario A, RF obtained the highest AUC (0.72), and in Scenario B, RF also reached the highest AUC (0.88). Consequently, Random Forest was selected as the representative model in both scenarios.

#### 2.4.4. Test-Set Performance and ROC Curves

**Figure 3:**
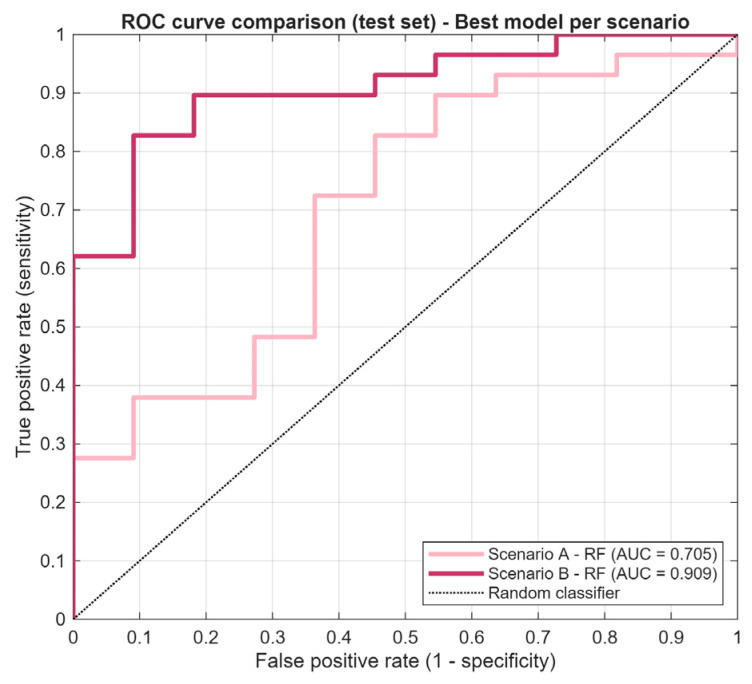
ROC curves of the Random Forest model on the test set.

The RF models were evaluated on the independent test set. Figure 3 shows the ROC curves on the test set, and Table 5 reports the metrics with their confidence intervals. Scenario A, without early-adherence information, reached an AUC of 0.71, with a sensitivity of 0.83 and a more limited specificity (0.45). Scenario B, which incorporates first-month adherence, reached an AUC of 0.91, a sensitivity of 0.90 and a specificity of 0.82. The comparison between scenarios shows the high predictive value of early adherence, suggesting that initial CPAP use is a relevant predictor of long-term adherence.

#### 2.4.5. Integration into a Clinical Decision Support Prototype

The model selected for each scenario was integrated into a web application prototype conceived as a proof of concept. Once the models were developed in MATLAB, the prototype was implemented in Python (version 3.13)—an open-source language that facilitates the implementation of the tool—using the Streamlit library, supported by the NumPy library for numerical computation and pandas. The application allows the patient’s variables to be entered and provides the adherence prediction estimated by the model, a final classification based on the decision threshold, and a set of indicative recommendations. It is demonstrative in nature and seeks to illustrate the clinical applicability of the models in a simple usage flow.

## 3. Case Study

**Figure 4:**
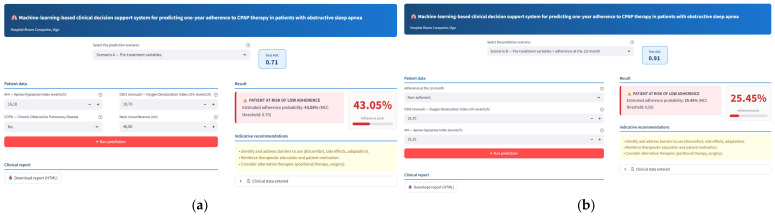
System output for the case study. (**a**) Scenario A prediction; (**b**) scenario B prediction.

To illustrate the operation of the system, a case based on a patient from the test set is presented: a 44-year-old man with moderate OSA and subsequent low adherence. The data entered are shown in Table 6. The patient was non-adherent in the first month—he used the device on more than 70% of nights, but without reaching 4 h on the days used—and maintained that pattern at one year. The Scenario A model estimated an adherence probability of 43.05% (threshold 0.70), classifying the patient as non-adherent already at the diagnostic stage, without any real-use data. The Scenario B model estimated 25.45% (threshold 0.59), reinforcing the classification: insufficient initial use acts as an early signal of discontinuation that the model captures, reducing the estimated probability from 43.05% to 25.45% (Figure 4). This case is included solely to illustrate the system’s usage flow and does not constitute additional validation evidence.

## 4. Discussion

### 4.1. Clinical Implications

The main contribution of this work is the possibility of identifying in advance the risk of low CPAP adherence, which would allow the intensity of follow-up to be personalized. In units that manage large patient portfolios (in the Vigo healthcare area alone, more than 10,000 active patients are estimated), equally intensive follow-up for all is unfeasible; the intelligent system developed in this work would make it possible to concentrate resources on the patients at greatest risk of discontinuation. Scenario A provides an estimate at the time of diagnosis (AUC 0.71; sensitivity 0.83) that is useful for guiding follow-up from the initial visit despite its lower specificity. Scenario B, which incorporates first-month adherence, improves overall performance (AUC 0.91; specificity 0.82) and is the most applicable in practice, since the first-month check is part of the usual protocols and telemonitoring is increasingly available [29,34]. The tool is conceived as support and not as a substitute for the professional, who integrates the prediction with contextual factors not captured by the model. It should be emphasized, however, that the low specificity of Scenario A (0.45) implies that a substantial proportion of truly adherent patients would be flagged for intensified follow-up (55 out of every 100), with the corresponding resource cost. For this reason, Scenario A is intended as an initial screening tool aimed at prioritizing sensitivity at the first visit, whose classification is re-assessed and refined with Scenario B at the first month, when specificity improves substantially (0.82) and the number of false positives decreases. To mitigate this cost, alternative operating points oriented toward higher specificity were also evaluated (requiring a minimum specificity of 60–70% on the training data), which allows the sensitivity–specificity balance to be adjusted according to the clinical and resource priorities of each unit.

### 4.2. Comparison with the State of the Art

Table 7 compares the results with representative works on CPAP adherence prediction using machine learning, restricted to studies that report AUC as the metric. Scenario A (AUC 0.71) lies in a range close to Scioscia et al. [35] (0.73), Eguchi et al. [36] (0.76) or Araujo et al. [37] (0.68–0.76), although these incorporate information from the initial follow-up, whereas Scenario A predicts using only pre-treatment variables. Scenario B (AUC 0.91) exceeds Gentina et al. [34] (0.87), lies above the range of Joymangul et al. [38] (0.80–0.87) and is consistent with Domínguez-Guerrero et al. [39], who in a cohort of 2180 patients reported an AUC of 0.91 at 3 months, 0.86 at 6 and 0.85 at 12; this comparison is especially relevant because both incorporate early device use, although its 12-month value (0.85)—a horizon equivalent to ours—invites caution in interpreting our figure given the smaller sample size. Along the same lines, Rafael-Palou et al. [40] built classifiers at different follow-up moments, with an F1-score of 0.73–0.76 under baseline conditions that rose to 0.82–0.87 when incorporating initial use, consistent with the jump observed between our Scenarios A and B (not included in Table 7 because it reports F1-score instead of AUC). Studies with larger sample sizes, such as Rusk et al. [41], achieve higher AUCs with deep neural networks under different methodological conditions; compared with them, the present work uses Random Forest models with few variables and a direct clinical interpretation. Overall, early-use information is a recurrent predictor, consistent with the review by Hanif et al. [29], and the fact that the priority variable in Scenario B was first-month adherence reinforces its predictive relevance.

### 4.3. Limitations

The study is single-center and lacks external validation on independent cohorts, which limits generalization. The sample size is small (200 patients; test set of 40), which is reflected in wide confidence intervals for some metrics. The models do not capture psychosocial, behavioral and contextual factors that the literature identifies as relevant predictors of adherence [21]. Finally, the prototype is demonstrative in nature and its functional and clinical validation in a real care setting remains pending. In particular, the test set included only 11 non-adherent patients, which explains the width of the confidence intervals (especially in Scenario A) and limits the stability of the estimates; likewise, no formal a priori sample-size calculation was performed. For all these reasons, this work should be interpreted as an exploratory, feasibility study; definitive confirmation therefore requires external validation in larger multicenter cohorts. Although 95% confidence intervals were obtained by percentile bootstrap, no additional optimism-corrected bootstrap estimate was calculated, which should be considered when interpreting the reported AUCs. In addition, using the same development dataset to perform feature selection and subsequently train the classifier is part of the standard model-development process and does not, by itself, introduce bias into the independent test evaluation. Importantly, the test set was not used during either RFE or classifier training. However, RFE was performed once on the complete 160-patient development dataset before cross-validation, which may introduce some optimistic bias into the cross-validation estimates because the feature-selection step was not repeated independently within each training fold. The independent test-set performance remains unaffected by this issue.

## 5. Conclusions

An intelligent clinical decision support system was developed and evaluated to predict one-year CPAP adherence in a cohort of 200 patients with OSA from Hospital Álvaro Cunqueiro in Vigo. The proposed objectives were met: a clinical database was structured for modeling, two prediction scenarios were defined, reduced subsets of variables were obtained, different algorithms were trained and compared, and the final model was integrated into a web prototype. Selection by RFE made it possible to obtain compact and interpretable models (Scenario A: AHI, ODI3%, COPD and neck circumference; Scenario B: first-month adherence, ODI3% and AHI), and Random Forest was the representative model in both. On the test set, Scenario A reached an AUC of 0.71 and Scenario B an AUC of 0.91, confirming that early adherence is a highly relevant predictor of one-year adherence.

The comparison with the literature shows that the system is competitive. Scenario A lies in the range of works that use initial follow-up information despite being based only on pre-treatment variables, and Scenario B reaches values comparable to those of studies with larger cohorts while keeping few variables and direct interpretability, although this comparison must be interpreted with caution given the small sample size and the single-center nature of the study. Future lines of work include validation in an external, multicenter cohort, the incorporation of tolerance variables and psychosocial factors, and the evaluation of the prototype through usability studies and, subsequently, prospective studies. In any case, this work should be understood as an exploratory, feasibility study (proof of concept): the results are preliminary and their performance requires confirmation through external, multicenter validation before any clinical application.

## Figures and Tables

**Table 1:** Descriptive summary of the main variables of the cohort (*n* = 200). Numeric variables as mean ± standard deviation; binary variables as percentage.

Variable	Type	Summary
Age (years)	Numeric	55.97 ± 12.11
BMI (kg/m^2^)	Numeric	33.46 ± 6.23
Epworth Sleepiness Scale	Numeric	10.81 ± 5.69
AHI (events/hour)	Numeric	41.72 ± 23.48
Supine AHI (events/hour)	Numeric	48.39 ± 27.22
Lateral AHI (events/hour)	Numeric	34.39 ± 26.44
Manual ODI3%	Numeric	44.05 ± 30.99
TC90 (%)	Numeric	23.28 ± 25.40
Number of obstructive apneas	Numeric	73.23 ± 101.79
Number of central apneas	Numeric	8.18 ± 18.19
Number of mixed apneas	Numeric	17.39 ± 41.46
Number of hypopneas	Numeric	186.35 ± 114.59
Initial CPAP pressure (cmH_2_O)	Numeric	7.57 ± 1.70
Neck circumference (cm)	Numeric	42.89 ± 4.65
Sex	Binary	Male: 75.00% (95% CI: 68.57–80.49%)
Professional driver	Binary	Yes: 6.50% (95% CI: 3.84–10.80%)
Alcohol consumption	Binary	Yes: 64.50% (95% CI: 57.65–70.80%)
Arterial hypertension	Binary	Yes: 48.50% (95% CI: 41.67–55.39%)
Stroke	Binary	Yes: 3.50% (95% CI: 1.71–7.05%)
Diabetes mellitus	Binary	Yes: 5.00% (95% CI: 2.74–8.96%)
Ischemic heart disease	Binary	Yes: 4.50% (95% CI: 2.39–8.33%)
Atrial fibrillation	Binary	Yes: 6.50% (95% CI: 3.84–10.80%)
Heart failure	Binary	Yes: 3.50% (95% CI: 1.71–7.05%)
COPD	Binary	Yes: 5.00% (95% CI: 2.74–8.96%)
Rhinitis	Binary	Yes: 16.50% (95% CI: 12.00–22.27%)
Depression	Binary	Yes: 12.00% (95% CI: 8.20–17.23%)
Hypnotic relaxants	Binary	Yes: 8.00% (95% CI: 4.98–12.60%)
Benzodiazepines (BZD)	Binary	Yes: 0.50% (95% CI: 0.09–2.78%)
Antidepressants	Binary	Yes: 0.50% (95% CI: 0.09–2.78%)
Antihistamines	Binary	Yes: 0.00% (95% CI: 0.00–1.88%)
High-intensity snoring	Binary	Yes: 39.50% (95% CI: 32.98–46.41%)
Choking awakenings	Binary	Yes: 17.00% (95% CI: 12.43–22.82%)
Multiple awakenings	Binary	Yes: 28.50% (95% CI: 22.69–35.12%)
Humidifier	Binary	Yes: 6.50% (95% CI: 3.84–10.80%)
CPAP mode	Binary	Yes: 98.50% (95% CI: 95.68–99.49%)
Work schedule	Multiclass	Exempt from work: 25.50%; No shift work: 58.00%; Shift work: 16.50%
Smoking status	Multiclass	Former smoker: 33.00%; No: 45.00%; Yes: 22.00%
Snoring	Multiclass	No: 3.00%; Yes: 92.50%; Only in supine position: 4.50%
Witnessed apneas	Multiclass	Daily: 15.50%; Sometimes (≤2 times/week): 37.00%; Frequently (>2 times/week): 14.50%; No: 33.00%
Intrasleep awakenings	Multiclass	Sometimes (≤2 times/week): 19.00%; Frequently (>2 times/week): 36.50%; No: 44.50%
Awakenings with shortness of breath	Multiclass	Sometimes (<2 times/week): 21.50%; Frequently (>2 times/week): 19.00%; No: 59.50%
Sleepiness while driving	Multiclass	At midday: 11.50%; On trips <30 min: 2.00%; On trips >30 min: 14.00%; No: 58.50%; Does not drive: 14.00%
Awakenings due to snoring	Multiclass	Sometimes (≤2 times/week): 10.50%; Frequently (>2 times/week): 2.00%; No: 87.50%
Nocturia	Multiclass	Sometimes (≤2 times/week): 48.00%; Frequently (>2 times/week): 26.50%; No: 25.50%
Vivid dreams	Multiclass	Sometimes (≤2 times/week): 43.50%; Frequently (>2 times/week): 18.50%; No: 38.00%
Non-restorative sleep	Multiclass	Daily: 40.00%; Sometimes (≤2 times/week): 26.50%; Frequently (>2 times/week): 13.00%; No: 20.50%
Morning grogginess	Multiclass	Sometimes (≤2 times/week): 30.50%; Frequently (>2 times/week): 33.50%; No: 36.00%
Daytime tiredness	Multiclass	Daily: 29.50%; Sometimes (≤2 times/week): 29.50%; Frequently (>2 times/week): 19.50%; No: 21.50%
Lack of concentration	Multiclass	Sometimes (≤2 times/week): 30.50%; Frequently (>2 times/week): 28.50%; No: 41.00%
Perceived sleepiness	Multiclass	Daily: 19.00%; Sometimes (≤2 times/week): 39.50%; Frequently (>2 times/week): 14.00%; No: 27.50%

**Table 2:** Distribution of one-year adherence after the stratified partition.

Subset	Adherent	Non-Adherent	Rate
Training (*n* = 160)	116	44	72.5%
Test (*n* = 40)	29	11	72.5%

**Table 3:** Predictor variables selected by RFE in each scenario, in order of importance.

Scenario	Selected Variables (in Order)
A	1. AHI 2. Manual ODI3% 3. COPD 4. Neck circumference
B	1. First-month adherence 2. Manual ODI3% 3. AHI

**Table 4:** Training and selection results. Mean cross-validation AUC and sensitivity/specificity with the MCC threshold. The selected model is shown in bold.

Scen.	Model	AUC	Sens.	Spec.	Optimal Hyperparameters
A	**RF**	**0.72**	**0.82**	**0.68**	trees = 300; MinLeaf = 20
A	LR	0.70	0.94	0.36	λ = 0.00113
A	SVM	0.71	0.96	0.43	C = 0.1; γ = 1
A	GB	0.69	0.84	0.73	it. = 100; lr = 0.05; depth = 3
B	**RF**	**0.88**	**0.86**	**0.73**	trees = 100; MinLeaf = 20
B	LR	0.84	0.80	0.84	λ = 0.00207
B	SVM	0.87	0.84	0.80	C = 0.01; γ = 0.1
B	GB	0.85	0.84	0.93	it. = 50; lr = 0.20; depth = 2

**Table 5:** Test-set results (*n* = 40) of the Random Forest model for each scenario, with 95% confidence intervals (bootstrap).

Metric	Scenario A	95% CI (A)	Scenario B	95% CI (B)
AUC	0.71	[0.52; 0.88]	0.91	[0.80; 0.99]
Sensitivity	0.83	[0.68; 0.96]	0.90	[0.77; 1.00]
Specificity	0.45	[0.15; 0.75]	0.82	[0.56; 1.00]

**Table 6:** Input data of the case-study patient.

Scenario	Variable	Value
A + B	AHI	16.10 events/h
A + B	Manual ODI3%	18.70 events/h
A	COPD	No
A	Neck circumference	46.00 cm
B	First-month adherence	Non-adherent (use <4 h/night or on <70% of nights during the first month)

**Table 7:** Comparison with representative state-of-the-art studies on CPAP adherence prediction using machine learning (studies that report AUC).

Study	N	Variables	Horizon	Algorithm	AUC
Gentina 2015 [34]	420	Baseline + CPAP days 1–15	4 months	Logistic reg.	0.87
Araujo 2019 [37]	3785	CPAP time series	30–150 days	Random Forest	0.68–0.76
Scioscia 2022 [35]	86	Baseline + polygraphy	12 months	SVM	0.73
Eguchi 2022 [36]	219	CPAP logs + demogr.	≈3 months	Logistic reg.	0.76
Joymangul 2023 [38]	1236	Quant. + qual. CPAP	30–90 days	NB/SVM	0.80–0.87
Rusk 2023 [41]	21,397	Daily use days 1–30	90–365 days	DNN	0.84–0.97
Domínguez-Guerrero 2026 [39]	2180	Baseline + PSG + CPAP 30 days	3–12 months	SVM/RF/MLP	0.85–0.91
Present (Sc. A)	200	Pre-treatment variables	12 months	Random Forest	0.71
Present (Sc. B)	200	Sc. A + 1st-month adher.	12 months	Random Forest	0.91

## Data Availability

The data presented in this study are available upon request from the corresponding author.

## Abbreviations

The following abbreviations are used in this manuscript:

AHI	Apnea-hypopnea index
AUC	Area under the curve
BMI	Body mass index
CDSS	Clinical decision support system
COPD	Chronic obstructive pulmonary disease
CPAP	Continuous positive airway pressure
CV	Cross-validation
GB	Gradient Boosting
LR	Logistic regression with Lasso penalization
MCC	Matthews correlation coefficient
ODI3%	3% oxygen desaturation index
OOB	Out-of-bag
OOF	Out-of-fold
OSA	Obstructive sleep apnea
RF	Random Forest
RFE	Recursive feature elimination
SVM	Support vector machine
TC90	Time with oxygen saturation < 90%
